# Panobinostat suppresses the mesenchymal phenotype in a novel claudin-low triple negative patient-derived breast cancer model

**DOI:** 10.18632/oncoscience.412

**Published:** 2018-04-29

**Authors:** Margarite D. Matossian, Hope E. Burks, Steven Elliott, Van T. Hoang, Annie C. Bowles, Rachel A. Sabol, Bruce A. Bunnell, Elizabeth C. Martin, Matthew E. Burow, Bridgette M. Collins-Burow

**Affiliations:** ^1^ Tulane University School of Medicine, Department of Medicine, Section of Hematology & Medical Oncology, New Orleans LA, USA; ^2^ Tulane Center for Stem Cell Research and Regenerative Medicine, New Orleans LA, USA; ^3^ Tulane University School of Medicine, Department of Pharmacology, New Orleans LA, USA; ^4^ Louisiana State University, Department of Agricultural Engineering, Baton Rouge LA, USA; ^5^ Tulane University School of Medicine, Tulane Cancer Center, New Orleans LA, USA

**Keywords:** triple-negative breast cancer, claudin-low, patient-derived xenograft, histone deacetylase inhibitor, extracellular matrix

## Abstract

Claudin-low triple negative breast cancer (CL-TNBC) is a clinically aggressive molecular TNBC subtype characterized by a propensity to metastasize, recur and acquire chemoresistance. CL-TNBC has a diverse intra- and extracellular composition and microenvironment, and currently there are no clinically approved targeted therapies. Histone deacetylase inhibitors (HDACi) have been investigated as therapeutic agents targeting invasive TNBC phenotypes. However, further studies are required to evaluate HDAC inhibition in CL-TNBC. Here, we utilize a novel CL- TNBC patient-derived xenograft model to study the various and diverse therapeutic potential targets within CL-TNBC tumors. To evaluate effects of the pan-HDACi panobinostat on metastasis and the mesenchymal phenotype of CL-TNBC, we utilize immunohistochemistry staining and qRT-PCR in *in vitro*, *ex vivo* and *in vivo* studies. Further, we evaluate pan-HDAC inhibition on stem-like subpopulations using 3D mammosphere culture techniques and quantification. Finally, we show that pan- HDACi suppresses collagen expression in CL-TNBC. In this study, we provide evidence that pan-HDAC inhibition has effects on various components of the CL-TNBC subtype, and we demonstrate the potential of our novel CL-TNBC PDX model in therapeutic discovery research.

## INTRODUCTION

Triple negative breast cancers (TNBCs) constitute approximately 12% of all breast cancers; TNBCs have a poorer short-term prognosis than other breast cancer subtypes [1]. There exist limited therapeutic options for TNBCs due to lack of expression of the commonly targeted estrogen receptor and HER2-receptor amplification. Histone deacetylases have multifunctional roles in oncogenesis, and have emerged as a potential therapeutic target in a number of tumor types, including breast cancer. HDAC inhibitors stabilize histone acetylation, affecting chromatin remodeling and epigenetics [2,3]. HDAC inhibition suppresses tumorigenesis and apoptosis of cancer cells, but has a minimal effect on normal tissue. HDAC inhibitors are in clinical trials of various cancer types, including T-cell lymphomas and multiple myeloma [3]. In breast cancer, HDAC inhibition demonstrates potent activity in an adjuvant setting with cytotoxic drugs, pro-drugs and ionizing radiation. Pan-HDAC inhibition is in phase I and II clinical trials as a combination treatment with an aromatase inhibitor for patients with metastatic breast cancer [4,5].

The majority of TNBCs are either of the basal- like (39-54%) or claudin-low (25 to 39%) molecular subtype classification [6]. These subtypes exhibit more aggressive clinicopathologic features compared to the more luminal-like molecular subtypes, demonstrated by the higher rates of metastasis, recurrence and chemoresistance [7]. Claudin-low (CL) TNBC is a primitive tumor with a complex microenvironment and diverse intratumoral heterogeneity [8,9]. This subtype is characterized by mesenchymal features, low expression of cell-cell junction proteins, and intense immune infiltrate [6]. In the CL-TNBC subset, expression of mesenchymal genes is correlated with higher levels of cancer stem-like cells designated as CD44+CD24-/low cells. Other studies have reported that CL-TNBC established cell lines (including MDA-MB-231) exhibit high populations of CD44+CD24-/low cells [10,11].

Established and immortalized cell lines have been used extensively in therapeutic discovery research both *in vitro* and *in vivo* to evaluate therapies' effects on cancer cells and tumorigenesis. However, a major limitation of these standard models is that they cannot accurately recapitulate molecular characteristics or behavior present in patient tumors. This is due, in part, to acquired mutations due to long-term cell culture, and lack of the complex microenvironment in the xenografted tumors, both on a cellular and 3D structural level. Xenografts derived from immortalized cell lines cannot form the 3D tumor structure created by extracellular matrix components that are present in human tumors. It is necessary to study all the components of a breast cancer subtypes as diverse and complex as the CL-TNBC subset in therapeutic discovery research. Utilizing patient- derived xenograft (PDX) models bypasses many of these limitations. PDX models are translational because in early passages they maintain intratumoral heterogeneity, genomic features and mutations present in the original patient tumor, as well as the extracellular matrix components unique to the original patient tumor [12-16]. Identification of novel targets is crucial in TNBC, and the extracellular matrix (ECM) has emerged in recent years as a therapeutic target [17]. Targeting the ECM may include targeting proteins that regulate the ECM, such as matrix metalloproteinases and lysyl oxidase, or structural components such as laminins, proteoglycans and collagen [18]. Since HDAC proteins modulate ECM composition and proteins that regulate ECM turnover, HDAC inhibitors have been investigated for their therapeutic potential to target the ECM in various diseases [19-22]. HDAC inhibition is significantly correlated with reduced tumor stage and depth of tumor invasion in gastric cancer [23]. However, the pathologic functions of HDACs in ECM regulation in breast cancer systems remains understudied. Further, extensive studies are required to evaluate the extent of HDACi in breast cancer ECM, and the specific mechanisms involved. Dissecting the various aspects of PDX breast tumors provides the ideal model to study how HDAC inhibitors, or other small molecule inhibitors, affect different microenvironment components in a translational setting. In our investigation, we utilize a novel CL-TNBC PDX model established in our laboratory [24].

HDAC inhibitors have biological effects in multiple cancer pathways, including proliferation, migration, angiogenesis and immune response [25]. For this reason, they have potential for targeting many individual components that comprise the complex CL-TNBC subtype, including targeting the mesenchymal phenotype, the cancer stem cell (CSC) population and extracellular matrix (ECM) components. Our laboratory has previously shown that LBH589 or panobinostat, a pan-DACi, reverses the mesenchymal cellular morphology and phenotype of TNBC cell lines [26]. Further, we observed that the more aggressive, highly proliferative behavior and invasive phenotype of TNBC cells render them particularly susceptible to the effects of panobinostat. However, this observation requires further investigation to be confirmed. Here, we utilize a translational CL-TNBC patient-derived xenograft model to demonstrate that pan-HDAC inhibition affects CL-TNBC.

## RESULTS

### Treatment of TNBC PDX explants with LBH589 recapitulates results observed in TNBC cell lines

**Figure 1:**
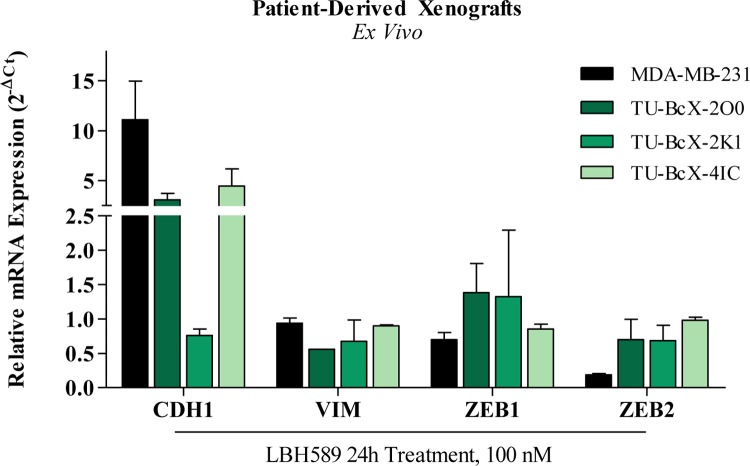
Treatment of TNBC PDX explants recapitulates results observed in TNBC cell lines *Ex vivo* treatments of TU-BcX-2O0 with LBH589 at 100nM for 72 hours. All data was obtained using qRT-PCR and normalized to actin and DMSO. Error bars represent SEM and significantly different ^*^p < 0.05, ^***^p < 0.001. TU-BcX-2O0 and TU-BcX-4IC was analyzed in duplicate, TU-BcX- 2K1 and MDA-MB-231 were analyzed in triplicate.

First, we evaluated if the effect of LBH589, a pan-DACi, had similar genomic changes after treatment of intact PDX tumors compared to our findings with treated cell lines. Rhodes et al. have previously shown that LBH589 significantly upregulates the epithelial marker *CDH1* and suppresses mesenchymal markers *VIM*, *ZEB1* and *ZEB2* mRNA expression in MDA-MB-231 cells [26]. Treatment of TU-BcX-2O0 explants, and to a lesser extent TU-BcX-2K1 and TU-BcX-4IC, with LBH589 recapitulated the genomic alterations observed in cell lines (increase in *CDH1* and reduced expression of *VIM* and *ZEB2*) (Figure 1). However, because genomic differences in TU-BcX- 2O0 treated explants most recapitulated cell line effects compared to our other TNBC models, we chose to interrogate TU-BcX-2O0 as the translational model in this study. We have previously described TU- BcX-2O0 as a claudin-low TNBC PDX model [24].

### *In vivo* treatment of TU-BcX-2O0 with LBH589 recapitulates *in vivo* observations

**Figure 2:**
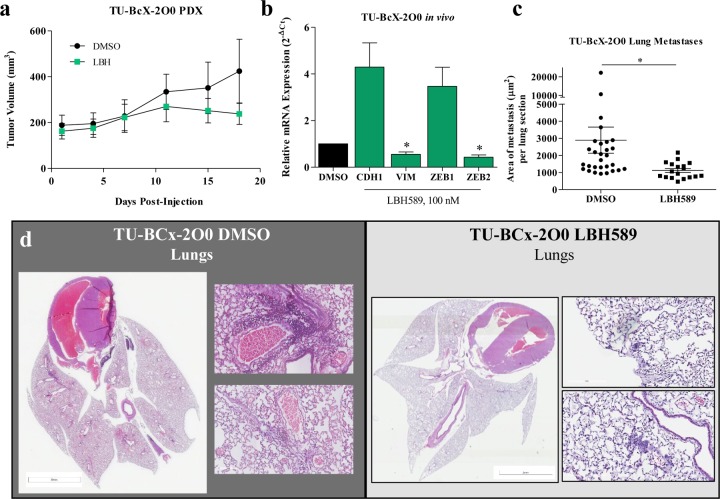
LBH589 suppresses tumorigenesis and metastasis *in vivo* using CL-TNBC PDX model **(A)** TU-BcX-2O0 tumors were implanted in the mfp of SCID/Beige mice and observed tumor growth. LBH589 (10 mg/kg i.p. injections; green data points) significantly suppressed tumorigenesis compared to DMSO vehicle control (black data points). **(B)** Analysis of *in vivo* treated tumors with qRT-PCR reveals an increase in CDH1 and significant reduction of VIM and ZEB2 mRNA expressions. All data was obtained using qRT-PCR in triplicate and normalized to actin. Error bars represent SEM and significantly different ^*^ p < 0.05, ^***^p < 0.001. **(C,D)** Lungs were harvested, fixed, embedded, sectioned and stained with H & E. Lung metastatic lesions were observed and quantified. LBH589 treatment significantly suppressed metastasis to the lungs compared to DMSO control.

We next performed an *in vivo* experiment utilizing TU-BcX-2O0 to show tumorigenic and metastatic effects of LBH589 in a translational model. Rhodes et al found that LBH589 suppressed tumor growth and metastases in mice xenografted with MDA-MB-231 cells [26]. Using our new PDX model, intact tumor pieces of TU-BcX- 2O0 were implanted in the mfp of SCID/Beige mice. After tumors formed, mice were normalized and treated with DMSO or LBH589 (Figure 2A). We confirmed our previous findings that LBH589 suppressed tumor growth *in vivo*. Next, we examined mRNA expression changes in the tumors removed at the termination of the experiment and found that, as observed in our *ex vivo* experiment and in cell lines, treatment with LBH589 increases *CDH1* and significantly suppressed *VIM* and *ZEB2* mRNA expression (Figure 2B). Finally, to examine metastatic effects of LBH589, lungs were harvested at the time of sacrifice. We found that LBH589 treatment significantly suppressed formation of lung metastases (Figure 2C-2D).

### LBH589 suppresses formation of TU-BcX-2O0 mammospheres

**Figure 3:**
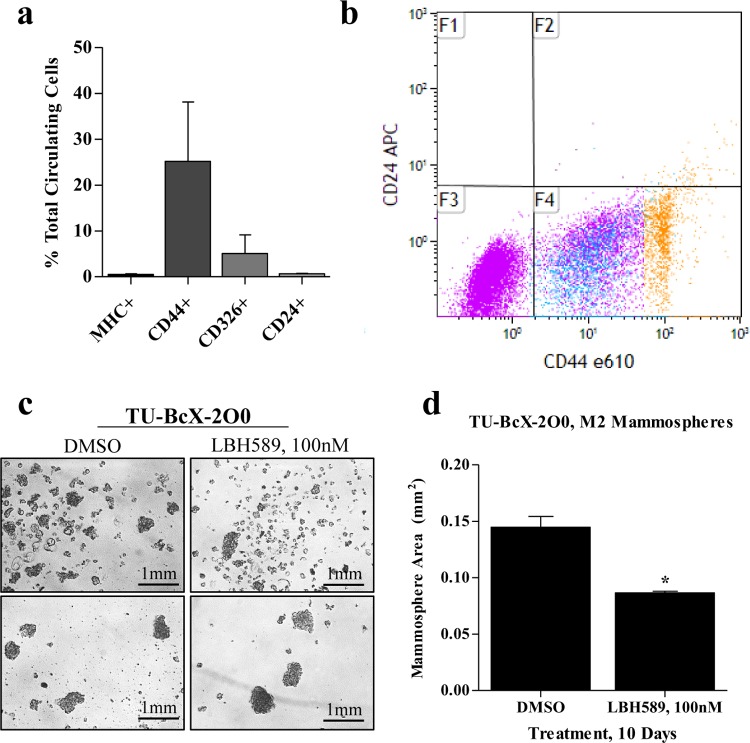
TU-BcX-2O0 has cancer stem cell-like characteristics and can be utilized in therapeutic discovery experiments **(A)** In circulating tumor cells isolated from SCID/Beige mice engrafted with TU-BcX-2O0, there is high percentage of CD44+ and a low percentage of CD24+ cells, indicative of a stem cell-like population. Furthermore, circulating cells also expressed CD326, or EpCAM, another stem cell-associated protein. Data was collected by flow cytometry, and protein expression is represented by percentage of total circulating cells. Data was collected in duplicate. **(B)** Flow cytometry dot plots of circulating tumor cells isolated from blood specimen of TU-BcX-2O0T5. Populations in blue are CD44low and populations in gold are CD44low. **(C,D)** Treatment of TU-BcX- 2O0 with LBH589 (100nM, 10 days) results in significantly reduced sizes of mammospheres. Error bars represent SEM and significantly different = ^*^p < 0.05, ^***^ p < 0.001.

Since claudin-low TNBCs have been shown to have high tumor initiating capability, exhibited by a high stem- cell like population, we first evaluated the presence of these populations in TU-BcX-2O0. *CD44*^hi^*CD24*^low ^cells reflect a stem cell-like population in breast cancer, capable of self-renewal and sphere formation in 3D culture. We observed *CD44*^hi^*CD24*^low ^populations in circulating cells isolated from the blood of the engrafted PDX mice at the time of TU-BcX-2O0 removal surgery, (Figure 3A-3B). These results show the circulating tumor cells harvested from TU-BcX-2O0 PDX models have distinct stem cell- like characteristics. Then, we plated TU-BcX-2O0 cells in 3D culture conditions; the plated cells formed stable spheres after 10 days, showing that the stem cell-like population can be enriched. We treated the next generation spheres with LBH589 (Figure 3C). Treatment with LBH589 for 10 days resulted in significantly reduced TU- BcX-2O0 sphere area compared to DMSO-treated control (Figure 3D). Due to low endogenous expression of *CDH1* and other adhesion proteins, TU-BcX-2O0 mammospheres formed as aggregates, similar to findings observed with MDA-MB-231 cells.

### LBH589 suppresses type I and increases type III collagen expression

**Figure 4:**
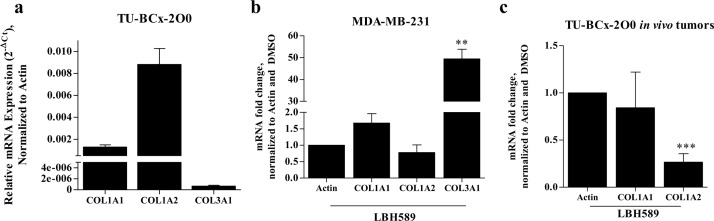
Collagen genes of TU-BcX-2O0 are altered by treatment with LBH589 **(A)** Baseline collagen genes (COL1A1, COL1A2 and COL3A1) were observed. TU-BcX-2O0 have higher levels of COL1A1 and COL1A2 compared to COL3A1. **(B)** MDA- MB-231 cells treated with LBH589 (100nM, 24h) show reduction of COL1A2 and increase of COL3A1 mRNA expression. **(C)** TU-BcX- 2O0 tumors treated with LBH589 from the *in vivo* treatment experiment have a significant reduction of COL1A2, similarly observed with MDA-MB-231 cells. COL3A1 mRNA expression were undetectable in all samples, and could not be analyzed. All data was obtained using qRT-PCR and normalized to actin. Error bars represent SEM and significantly different ^*^ p < 0.05, ^***^ p < 0.001. Data from tumors was performed in quadruplicate; all other data was performed in triplicate.

The complex extracellular matrix composition of TNBC tumors also contribute to acquisition of an invasive phenotype. Type III collagens (*COL3A1*) are associated with more benign, non-invasive breast cancers, and expression of type I collagens (*COL1A1*) are associated with invasive phenotypes [27,28]. Type III collagens direct stromal organization and limit metastases [29]. We found that LBH589 treatment of intact TU-BcX-2O0 tumors reduced *COL1A2* and increased *COL3A1* mRNA expressions. First, we show that at baseline, TU-BcX-2O0 has higher mRNA expression of type I collagens, *COL1A2* and *COL1A1*, and lower expression of *COL3A1* (Figure 4A). In MDA-MB-231 cells treated with LBH589, we observed reduced expression of *COL1A2* and increased expression of *COL3A1* (Figure 4B). We observed a similar reduction of *COL1A2* expression in TU-BcX-2O0 *in vivo* tumors. However, *COL3A1* mRNA expression levels were too low (undetectable) by qRT-PCR in the *in vivo* samples, and data was not reported. COL1A1 gene expression changes were not significant in either MDA-MB-231 nor TU-BcX-2O0 cells.

## DISCUSSION

In this study, we demonstrate the value of using translational PDX models in target discovery and therapeutic research, especially in clinically aggressive TNBC tumors such as the claudin-low TNBC subtype. For these studies, we utilized a small molecule inhibitor that our laboratory has extensively studied in TNBC systems, LBH589 or panobinostat, a pan-DACi. We observed that treatment of various TNBC PDX explants, established in our laboratory, with LBH59 has similar gene expression changes that are characteristic of mesenchymal phenotype reversal (increase in *CDH1*, decrease in *VIM* and *ZEB2*). Notably, we show that these changes are recapitulated more closely in the TU-BcX-2O0 CL-TNBC PDX model than in the TU-BcX-2K1 or TU-BcX-4IC models , and chose to focus on the effects of LBH589 on the CL-TNBC model.

In an *in vivo* study utilizing the TU-BCx-2O0 model, we evaluated if our previous *in vivo* observations were recapitulated [26]. We found that LBH589 suppresses tumorigenesis and metastasis in TNBC xenografts, especially in xenografts derived from CL-TNBC cell lines including MDA-MB-231. LBH589 suppressed tumorigenesis of TU-BcX-2O0 significantly, and significantly reduced number and area of lung metastases. Additionally, LBH589 had the same effects on gene expression that were observed in both the PDX explants and in the cell lines (increase in *CDH1*, reduction of *VIM* and *ZEB2*).

The CSC population within TNBCs and other solid tumor types is another cell population to target with therapeutics that PDX models can be used to evaluate. The relative density of cancer stem cell populations within tumors is correlated with metastatic potential and therapeutic resistance [30,31]. TU-BcX-2O0 explants were plated in reduced growth factor media in ultra-low attachment flasks and observed sphere formation over 10 days. We found the TU-BcX-2O0 forms spheres under these conditions, suggesting evidence of CSC populations. However, the low endogenous expression of claudins and other adhesion proteins causes the spheres to form in aggregates, and not as true spheres as visible in another TNBC PDX we established, TU-BcX-2K1. We analyzed the peripheral blood harvested from the passaged mice using flow cytometry to observe expression of stem cell- like populations in circulating cells in the TU-BcX-2O0 xenografted mice. With co-expression staining based on flow cytometry data, we found a detectable population of *CD44*+/*CD24*low tumor cells in circulation. We tested the practicality of this application of PDX models using LBH589. In concordance with other studies in the literature, LBH589 suppressed the area of TU-BcX- 2O0 mammospheres, and reduces effects of the CSC population.

The extracellular matrix is another aspect of the tumor microenvironment that has been investigated as a potential therapeutic target, specifically the collagen networks that surround tumor cells. Although collagens have a diverse role in breast cancer tumorigenesis and metastasis, two collagen subtypes have been identified to predict breast cancer invasiveness. Collagen type I is associated with increased invasiveness and recruitment of stromal cells and other microenvironment components and is increased in metastatic subtypes. Conversely, collagen type III suppresses invasive potential of breast cancer cells. TU-BcX-2O0 has higher endogenous expression of COL1A1 and *COL1A2*, and lower mRNA expression of *COL3A1*. HDAC inhibitors have been investigated to suppress collagen formation and synthesis in various pathologies [19,22,32]. After treatment with LBH589 in MDA-MB-231, we observed a reduction of *COL1A2* and a significant increase in *COL3A1*, suggesting a switch from the invasive collagen expression to collagen types that suppress the invasive potential. In TU-BcX-2O0 we also observed a significant reduction in *COL1A2* mRNA expression, although *COL3A1* expression changes could not be evaluated because the relative cycle threshold values of *COL3A1* were too low to be quantified in our qRT-PCR system.

Claudin-low TNBC exhibits aggressive clinicopathologic features; this molecular subtype of TNBC has higher propensity to metastasize, recur, and acquire chemoresistance. Further, due to the intratumoral heterogeneity and diverse representation of various cell populations within this tumor subtype, discovery of a targeted therapy remains evasive. To investigate potential therapies, more translational models are required to study various targetable components within a tumor: the cancer cells, the CSC subpopulation, the tumor microenvironment and the extracellular matrix of the tumor. Our laboratory previously determined that HDACi exhibited higher efficacy in more aggressive TNBC types, including CL- TNBC. We utilized a translational PDX model to evaluate the role of pan-HDACi on the various therapeutic targets within the CL-TNBC tumor.

In this study, we show that LBH489 suppresses tumorigenesis and metastasis and reduces mesenchymal gene expression and increases epithelial gene expression in a CL-TNBC PDX model. These results are consistent with previous results using established cell lines, demonstrating the utility of our CL-TNBC PDX model in therapeutic discovery research. Further, we show LBH589 suppresses cancer stem cell populations and alters the extracellular matrix/collagen composition of TU-BcX-2O0. However, further studies are required to fully characterize the specific mechanisms of LBH589 in collagen remodeling.

## MATERIALS AND METHODS

### Reagents

Dulbecco's modified Eagle's medium (DMEM), Dulbecco's phosphate-buffered saline (PBS), phenol- red free DMEM, fetal bovine serum (FBS), essential amino acids, non-essential amino acids (NEAA), antibiotic/anti-mitotic, penicillin/streptomycin, sodium pyruvate, and EDTA (0.5 M, pH8) were obtained from GIBCO (Invitrogen; Carlsbad CA, USA). Insulin was purchased from Sigma-Aldrich (St. Louis MO, USA) and charcoal stripped FBS from HyClone (Thermo Scientific; Logan UT, USA). Dimethyl sulfoxide (DMSO) was obtained from Research Organics, Inc (Cleveland OH, USA).

### Cell Culture

Human MDA-MB-231 cells were obtained from the American Type Culture Collection (ATCC, Manassas, VA, USA) and are characterized as triple-negative/basal B mammary carcinoma. Cells were maintained in DMEM supplemented with 10% FBS, NEAA, MEM amino acids, anti-anti (100 U/mL), sodium pyruvate and porcine insulin (1 x 10-10 mol/L) at 37°C in humidified 5% CO For treatment studies, cells were grown in phenol red-free DMEM supplemented with 5% charcoal-stripped FBS and supplemented with NEAA, MEM amino acids, Gluta-Max and penicillin (100 U/mL).

### Patient-derived xenografts

*SCID/Beige (*CB17.Cg-*PrkdcscidLyst bg*/Crl*)* were purchased from Charles River and are used to prevent rejection of the xenografted human tumors. The autosomal recessive SCID (Prkdcscid) mutation results in severe combined immunodeficiency affecting both the B and T lymphocytes. The autosomal recessive beige (Lystbg) mutation results in defective natural killer (NK) cells. Tumor tissues from each patient were cut into 3 x 3 mm2 pieces under aseptic sterile conditions, coated with full factor Matrigel (Cat No. 354234, Fisher Scientific, Waltham, MA, USA) and implanted bilaterally into the mammary fat pads (mfp) of mice under isoflurane and oxygen. Tumors were measured using a digital caliper after ostensible tumor take was established. Tumors were passaged when tumor volume achieved 750-1000 mm3. To passage, mice were euthanized by CO2 and tumors were removed, dissected, coated in full factor Matrigel, and then implanted bilaterally into new mice that were anesthetized using a mix of isoflurane and oxygen delivered by mask. Before surgery, mice were given Meloxicam (5 mg/kg/day, for 3 days' post-surgery) for pain. For *in vivo* treatments, tumor pieces (3 x 3 mm2) were engrafted bilaterally in the mfp of SCID/Beige mice (5/group). When tumors became measurable, mice were injected intraperitoneally (i.p.) with vehicle (DMSO) or LBH589 (10mg/kg) every three days. For *ex vivo* treatments tumor pieces were plated in 24-well adherent plates and covered with DMEM media. Tumors were treated in-well with control (DMSO) or LBH589 (1µM).

### RNA extraction and quantitative real-time PCR Analysis

Explants were collected after 72 hours, and RNA was extracted using QIAzol Lysis Reagent (Cat No. 79306; Qiagen, Valencia, CA, USA) and dissection of the tumor with scissors. Total RNA (2 µg) was reverse- transcribed (iScript kit, BioRad Laboratories, Hercules, CA, USA) and analyzed by qRT-PCR. All qRT-PCR data were normalized to actin. Primer sequences are as follows (Invitrogen, Carlsbad, CA, USA). *COL3A1, COL1A2,* and *COL1A1* primers were ordered from Integrated DNA Technologies (Coralville, IA, USA). *β-actin* F--5'- GGCACCCAGCACAATGAAGA-3'; *β-actin* R-5'- ACTCCTGCTTGCTGATCCAC -3'; *CDH1* F-5'-AGGTGACAGAGCCTCTGGATAGA-3', *CDH1* R-3'-TGGATGACACAGCGTGAGAGA-3' *FOXA1* F-5'-ACTCCAGCCTCCTCAACTGCG-3', R-5'-GTGCCAAGCCGTGTGCCG-3'; *ZEB2* F-5'- TGCACTGAGTGTGGAAAAGC-3', *ZEB2* R-5'- TGGTGATGCTGAAAGAGACG-3'; *COL3A1* F-5'-TGCCCTACTGGTCCTCAGAA-3', *COL3A1* R-5'-TGCGAGTCCTCCTACTGCTA-3'; *COL1A1* F-5'-CAGCCGCTTCACCTACAG-3', *COL1A1* R-5'- TTTTGTATTCAATCACTGTCTTGCC-3'; *COL1A2* F-5'-CATTAGGGGTCACAATGGTC-3', *COL1A2* R-5'-TGGAGTTCCATTTTCACCAG-3'. qRT-PCR was conducted as previously published [25]. Data were represented as normalized fold expression compared with DMSO control of biological triplicate samples ± SEM.

### Flow Cytometry and Fluorescence Activated Cell Sorting (FACS)

To analyze CSC phenotypes, TU-BcX-2O0 was enzymatically digested with type I collagenase (Worthington Biochemical Corporation, Lakewood, NJ, USA) at room temperature, neutralized with media, and then filtered. Circulating tumor cells were collected in whole blood with 0.5M EDTA (Gibco Invitrogen, Carlsbad CA, USA), incubated in red blood cell lysis buffer (0.008% NH4Cl, pH 7.2-7.4; Sigma-Aldrich, St. Louis MO, USA) and washed with PBS. Collected cells from the tumor and blood samples were placed in staining solution containing 1% Bovine Serum Albumin (BSA; Sigma- Aldrich) and 1% *CD16/CD32* Mouse BD Fc BlockTM (BD Biosciences) in PBS. The following primary antibodies were used: Anti-human *CD24* (APC), anti-human *CD326* (EpCAM; PerCP-eFluor710) and anti-human/mouse *CD44* (PE-Fluor 610) purchased from eBiosciences (San Diego, CA, USA). All cells from the blood were analyzed with a Galios Flow Cytometer (Beckman Coulter, Brea, CA, USA) running Kaluza software (Beckman Coulter). At least 5000 events were analyzed and reported as the mean ± SEM.

### PDX Decellularization and Histological Analysis

PDX tumors were decellularized through a modified protocol previously described [33,34]. In brief, tumor samples were collected and left in water for 24 hours at 4°C followed by incubation in Triton X-100 (Bio-Rad, Hercules CA, Cat. No. 1610406). Cells were first washed in deionized water and incubated in sodium deoxycholate solution (ThermoFisher Scientific, Waltham MA, USA), then washed in deionized water and incubated in calcium chloride (Sigma-Aldrich, St. Louis MO, USA) and finally washed with deionized water ad treated with DNAse I (1 U/mL, Sigma-Aldrich, St. Louis MO, USA) and antibiotic-antimycotic (100 U/ml; ThermoFisher Scientific, Waltham MA, USA) solutions. Decellularized tumors were then formalin-fixed (10%), paraffin-embedded and sectioned for Picosirius Red staining. Total collagen content was observed through 4X objective on an Olympus BX51 system microscope. Analysis of collagen fibers was performed in Matlab with a custom code. Quantification of collagen I:III and subsequent representative darkfield images were obtained through use of polarized light microscopy, as described previously [35].

### Mammosphere Culture

Mammospheres were cultured by plating explants in 3D culture composed of DMEM/F-12 media supplemented with B-27, penicillin- streptomycin, fibroblast-growth factor and epidermal-growth factor (Invitrogen, Carlsbad, CA, USA). Explants were plated in low-attachment 6-well plates (ThermoFisher Scientific, Waltham MA, USA) and primary spheres grew for up to 10 days before harvesting and quantified. Sphere area was quantified in the Aperio program.

### Statistical analysis

Studies run in triplicate were analyzed by unpaired Student's *t-*test (Graph Pad Prism V.4). *p-*Values < 0.05 were considered statistically significant. The flow cytometry analysis of the circulating tumor cells was run in duplicate; all other analyses were run in triplicate.

## Abbreviations

CL: claudin-low
TNBC: triple negative breast cancer
PDX: patient-derived xenograft
HDACi: histone deacetylase inhibitor
CDH1: E-cadherin
VIM: vimentin
CDH2: N-cadherin
DMSO: dimethyl sulfoxide
